# The experienced positive and negative influence of HIV on quality of life of people with HIV and vulnerable to HIV in the Netherlands

**DOI:** 10.1038/s41598-022-25113-5

**Published:** 2022-12-19

**Authors:** Kim A. G. J. Romijnders, Laura de Groot, Sigrid C. J. M. Vervoort, Maartje Basten, Berend J. van Welzen, Mirjam E. Kretzschmar, Peter Reiss, Udi Davidovich, Maarten F. Schim van der Loeff, Ganna Rozhnova

**Affiliations:** 1 Julius Center for Health Sciences and Primary Care, University Medical Center Utrecht, Utrecht University, Utrecht, The Netherlands; 2 Department of Internal Medicine, University Medical Center Utrecht, Utrecht, The Netherlands; 3 Amsterdam UMC location University of Amsterdam, Global Health, Amsterdam, The Netherlands; 4 Amsterdam Institute for Global Health and Development, Amsterdam, The Netherlands; 5 Amsterdam UMC location University of Amsterdam, Infectious Diseases, Amsterdam, The Netherlands; 6 Amsterdam Institute for Infection and Immunity, Amsterdam, The Netherlands; 7 Department of Social Psychology, University of Amsterdam, Amsterdam, The Netherlands; 8 Department of Infectious Diseases, Public Health Service of Amsterdam, Amsterdam, The Netherlands; 9 BioISI – Biosystems & Integrative Sciences Institute, Faculdade de Ciências, Universidade de Lisboa, Lisbon, Portugal; 10 Center for Complex Systems Studies (CCSS), Utrecht University, Utrecht, The Netherlands

**Keywords:** HIV infections, Risk factors, Preventive medicine, Quality of life

## Abstract

This qualitative study aimed to explore the experienced influence of HIV on the quality of life (QoL) of people with HIV (PHIV) and key populations without but are vulnerable to HIV in the Netherlands. We conducted and thematically analyzed interviews with 29 PHIV and 13 participants from key populations without HIV (i.e., men who have sex with men). PHIV and key populations shared positive meaningful experiences regarding HIV, i.e., feeling grateful for ART, life, and the availability of PrEP, being loved and supported in the light of HIV, and providing support to the community. Negative predominant experiences regarding HIV were described by both PHIV and key populations as the negative effects of ART, challenges with regards to disclosing HIV, social stigmatization, and self-stigma. It remains important to support HIV community organizations in their efforts to reduce social stigmatization and to continue improving biomedical interventions for HIV.

## Introduction

The perspective of HIV has improved dramatically over the years, changing from a fatal disease to a manageable chronic condition^1^. This paradigm shift was driven by the introduction and ongoing development of antiretroviral therapy (ART) and improved treatment strategies^2^. Furthermore, the use of pre-exposure prophylaxis (PrEP) and undetectable equals untransmittable (U = U) revolutionized biomedical interventions for HIV prevention^3–5^. Despite these improvements, there is a need for continued attention to the experienced influence of HIV on the quality of life (QoL) of people with HIV (PHIV) and key populations who are living without but vulnerable to HIV (e.g., men who have sex with men (MSM) and ethnic minorities). Evidence suggests that both groups currently continue to experience a multifaceted and predominant influence of HIV on their QoL^6^. This influence may have resulted in lower experienced QoL among PHIV and key populations compared to the general population^4,6,7^. Several studies demonstrated a predominant influence or burden of HIV and of ART on the QoL of PHIV and key populations in over 25 countries, including the Netherlands, the USA, and Australia^3,5,7–9^. For example, being dependent on ART was shown to be a cause for emotional and physical discomfort among PHIV^3,5,7,8^.

Removing the multifaceted and predominant influence of HIV on the lives of PHIV and key populations is desired and strived for by clinicians, biomedical researchers, and patient organizations^6,10–13^. Most research has focused on improving the clinical management of HIV (i.e., diagnosis, treatment, viral suppression etc.) to reduce this predominant influence^3,5,7–9^. Several studies underlined that shifting the focus beyond the clinical management of HIV will ultimately improve the QoL^4,6,7,13,14^. Some studies addressed the multifaceted influence beyond exploring the experienced negative predominant influence of HIV^15–21^. For example, Herron et al.^16^ reported in a review of qualitative literature that women with HIV experienced stigma, socio-structural barriers to healthcare and support, and negative encounters with health professionals due to HIV. However, they also found that women with HIV experienced positive personal growth due to HIV. We aimed to investigate the influence of HIV on the QoL in all its breadth including both the positive and negative influence of HIV that have received little attention in the literature. In addition, few studies addressed the influence of HIV among key populations who are vulnerable to but are not living with HIV. Koester et al.^22^ demonstrated that PrEP reduced the fear of HIV acquisition among key populations. However, to the best of our knowledge, all aspects of the experienced influence of HIV on the QoL of key populations specifically have not been studied. The inclusion of key populations, when investigating this influence, is important to direct the research community towards prevention interventions that fit the needs of people vulnerable to HIV^4,6,7,10–14,23^.

Our qualitative study aims to explore the experienced influence of HIV on the QoL of PHIV and key populations vulnerable to HIV in the Netherlands. In the context of this study, key populations are people injecting drugs and MSM living without HIV. This study reports findings from the secondary analysis of data collected in a research project that explored the current influence of HIV on the QoL and perceptions towards an HIV cure^6^ among PHIV and key populations in the Netherlands.

## Methods

This qualitative study describes the results of in-depth interviews exploring the influence of HIV on the QoL of PHIV and key populations in the Netherlands. Consolidated criteria for reporting qualitative studies (COREQ)^24^ are reported in Supplementary Table I.

### Study population

Participants were eligible if they were a Dutch or English-speaking adult (≥ 18 years), living with HIV, or belonging to key populations. To ensure variation in participants’ characteristics, PHIV and participants from key populations were purposefully sampled from October 2020 till March 2021 from the Amsterdam Cohort Studies (ACS)^25^, the AGE_h_IV Cohort Study^26^, the infectious diseases outpatient clinic of the UMC Utrecht, and the Dutch HIV Association^27^. The sampling was scheduled to stop after inductive thematic saturation had been reached^28^.

### Data collection

Semi-structured in-depth interviews were conducted online using WebEx (n = 31) or face-to-face at the UMC Utrecht (n = 11), depending on the participant’s preference. Participants received reimbursement for their time (€12, 50) and travel expenses. An interview topic guide was used (Table 1) to explore the current influence of HIV on the QoL of PHIV and key populations vulnerable to HIV. All in-depth interviews were conducted by the first author (Ph.D.), an academic health behavioral scientist. The in-depth interviews started with an introduction of the study and the interviewer and were followed by an explanation of the set-up of the interviews. Informed consent was orally discussed and signed by participants. Subsequently, information about socio-demographic characteristics of participants was collected. During the interviews, open-ended questions were used to stimulate participants’ own interpretation. Prompts were used to further encourage deliberation. Notes were taken during the interviews to describe nonverbal communication such as bodily movements and facial expressions. The interviews were conducted in either Dutch (n = 37) or English (n = 5) and lasted 45 to 90 min.

**Table 1:** Interview topic guide for PHIV and key populations.

Topic	Example questions or description
Introduction interview	Interviewer introduced the interviews
Informed consent	Verbal and written consent was provided
**Experienced influence of HIV**	
Influence of HIV	“What is the role of HIV in your life?”
	“What did you think of HIV before you had HIV?”
	“What is your perception of HIV?”
	“What is your opinion of ART/HIV medication?”
	“How does HIV influence your quality of life?”
Quality of life	“How would you describe your quality of life?”
	“What provides you with quality in life?”
	*Prompt how HIV influences this quality*
	“How does [insert aspect mentioned] provide you quality of life?”
	*Prompt how HIV influences [mentioned aspect]*
	“How did HIV change your quality of life?”
Closing	Closing of the interview

### Data analyses

The in-depth interviews were conducted in a cycle of six interviews and transcribed verbatim^29^. The transcribed interviews of one cycle (i.e., consisting of 6 in-depth interviews) were assessed for inductive thematic saturation^28^. The interpretative thematic data analysis was peer reviewed by an expert in qualitative research [S.C.J.M.V] and consisted of six stages (Supplementary Table II)^30,31^. These stages enabled us to identify recurring topics, ideas, and patterns within our data. Inductive thematic saturation was reached after cycle 6^28^. NVivo Version 12 supported the analysis^32^. In addition, the forward–backward method was used by K.A.G.J.R and L.d.G. to translate Dutch quotes into English. To enhance the reliability of the data analysis, we used the 15-item checklist by Braun and Clark^30^ to consider the quality of our thematic analysis.

### Ethical approval

The study was approved by the ethics committee of the University Medical Center Utrecht (UMCU): 20-546/C.

## Results

Of the 44 scheduled interviews, 42 were conducted. Twenty-nine of the participants had HIV, and 13 belonged to key populations. Nine of the 42 participants were women, of whom one identified as a transwoman, and ages ranged from 24 to 72 years. Overall, many PHIV and participants without HIV shared experiences about the multifaceted influence of HIV on their QoL that were described as predominant and negative or positive and meaningful. No differences were observed in the response or interaction between the online and face-to-face interviews.

### Positive meaningful experiences of HIV on QoL

Both PHIV and participants from key populations described meaningful experiences related to HIV that were perceived as positive (Table 2).

**Table 2:** Quotes supporting the positive meaningful experiences of HIV on QoL.

Theme	Sub-theme	Quote #	Quote
Positive meaningful experiences of HIV on QoL	Appreciating life & health	1	Well, I think having HIV has been—in a way—a blessing. Don’t ask me [to share this with others…] I will never say that to anyone. But it has made me pause and look at life differently… Having HIV basically made me look at life differently… [female, 47, bisexual, living with HIV for more than 20 years]
	Appreciating life & health	2	If I love myself, I don’t have to think twice about taking my HIV medication. And making sure that I live… So, I think it all comes down to love for myself … Only love will make you realize such things, good and bad, and critical… I think it [love for myself] grew more, I think, HIV made me respect and appreciate life and love even more… and even realize the depth of love even more. You know how unconditional it can be and how non-judgmental it can be. [female, 42, heterosexual, living with HIV for more than 20 years]
	Appreciating life & health	3	I’m fitter than the majority of people who are HIV negative. Because I train, I look after myself. I watch what I eat, I understand my diet. I see people I work with and if I see I’m trying to run up a flight of stairs and they’re completely destroyed by it and you think, yeah, I’ve just run up there and I'm carrying something on my shoulder. What’s your problem? They see me as… they don’t see me as a person with the disease [HIV] [male, 54, gay, living with HIV for 16 years]
	Social support and sense of belonging	4	I told everybody… Like a band aid, I ripped it off and told everybody immediately… For me that was the best way, that felt really nice… Sometimes I need a hug and I get a hug, sometimes I don’t want to talk about it, and they leave me alone… My dad, he is very sensitive, for a moment he thought I would die. He took a big hit. My mom is very matter of fact. She asked a lot of questions about what it means, what the next steps were… information was a relief. [male, 33, gay, living with HIV for 4 years]
	Social support and sense of belonging	5	The HIV community is a very close-knit community and I really feel a part of that. I also have the idea that I can and do something really meaningful for it. You know, by standing up for the rights of people living with HIV, fighting stigma with my face in the newspaper, and telling you that you can live a normal life with HIV. Basically, educating people… in that community I feel very much at home, and I have found something meaningful in life… My activism is also something that drives me, what defines me… But it’s definitely a very special community, that's for sure… I’ve always said, being able to do something with something bad that happened to you… that’s just a very nice thing. Then I’m useful, or meaningful or something. [female, 58, heterosexual, living with HIV for more than 20 years]
	Appreciation of the lifeline that ART offers	6	It’s a privilege because I have access to medication. I’m very fortunate that I live in the Netherlands and that I live now and not 20 or 30 years ago. That would have been a different story. Then I wouldn’t have been here anymore. [male, 38, bisexual, living with HIV for 6 years]
	Managing the predominant influence of HIV	7	It’s not a dirty little secret anymore. [male, 54, gay, living with HIV for 16 years]
	Managing the predominant influence of HIV	8	That’s [U = U] what I'm starting with: “I have a virus and I can’t transmit it, but the virus is HIV.” And that’s where I start, and then I’ll be silent for a while, and then it’s up to them to process that. I need to explain that I take medication, that it is a chronic illness, that I cannot infect anyone, and that there have been a lot of medical developments. People just don’t know. I mean, I didn’t know it before I got infected either. I’ve had to educate myself, so people who hear about it [HIV] need to be educated too. [male, 45, heterosexual, living with HIV for 3 years]
	Managing the predominant influence of HIV	9	PrEP… I take it intermittently. I’m so glad it [PrEP] exits. It totally changed sex. It is so much freer now. In the back of your head you were always thinking: I could acquire HIV… If you grew up with this thing [HIV] hanging over your head… that does something to a person [PrEP changed sex] [male, 56, gay]

#### Appreciating life & health

Several participants with HIV explained that HIV confronted them with their own mortality. They described that this conformation made them appreciate life more and affected what added quality to their life (Table 2*Quote 1*). The realization about what is currently important for the QoL of participants with HIV grew due to HIV and was often linked to love and being loved by others (Table 2*Quote 2*). Many PHIV also indicated to appreciate their health, physical strength, and capabilities even more due to HIV. They shared that because they were grateful of their body fighting HIV, they took great pride in taking good care of their body (Table 2*Quote 3*).

#### Social support and sense of belonging

Some PHIV described to feel supported after they disclosed their HIV status to family, friends, and steady partners (Table 2*Quote 4*). Being part of a community was also described as a source of support for many participants with HIV. This community provided PHIV with a sense of belonging to something greater than themselves. Being part of the HIV community was explained as not only receiving support, but also providing support and providing a meaningful contribution (Table 2*Quote 5*).

#### Appreciation of the lifeline that ART offers

Participants, whether they had or did not have HIV, expressed to be grateful to be living in the times when there is effective treatment for HIV. Participants were aware that ART provides a lifeline. They were able to compare their situation to that of other people and explained the dependency on ART in a positive way. This let some interviewees to gratefully compare their current situation to regions of the world where ART is not easily available (Table 2*Quote 6*).

#### Managing the predominant influence of HIV

The evidence and experience with U = U and PrEP helped both PHIV and key populations to manage the predominant influence of HIV. PHIV explained that U = U helped them to feel better about themselves and reduced their self-stigma because it was scientifically proven that they were not infectious if they adhered to their medication (Table 2*Quote 7*). In addition, many participants described positive meaningful experiences related to U = U because it made explaining HIV to others easier (Table 2*Quote 8*). Some participants of both participants groups perceived PrEP as a way of preventing HIV and dealing with the fear of contracting HIV during sex with or without using condoms. This way, PrEP was explained to free a user and a sex partner with HIV of the control HIV has on the sex life of PHIV and key populations (Table 2*Quote 9*).

### Life dominated by HIV

Some PHIV and participants from key populations explained the constant influence of HIV on their QoL as multifaceted and predominated (Table 3). The following sub-themes represent the facets of this influence (Table 3*Quote 10*).

**Table 3:** Quotes supporting how life is dominated by HIV.

Theme	Sub-theme	Quote #	Quote
Life dominated by HIV		10	Due to HIV, my life is no longer… free. I will always be part of a group. There are so many emotions connected to it [HIV]. My… values were oppressed by my [HIV] diagnosis. It became harder for me to reach and to connect with people. I closed myself off. I became isolated. I became very depressed and had suicidal thoughts. That is why my values [respect, connection, empathy, kindness, and being active] are so important for me. Because those values were oppressed [by HIV]. Yes, HIV made me give up on everything on the short and long term. [I needed to find] my resilience. I decided to go after it [resilience] again [male, 38, bisexual, living with HIV for 6 years]
	Being constantly dependent on ART	11	If I went to someone’s home for a nice dinner and I’m alone and I drink a couple of glasses of wine… Then I can’t stay over spontaneously, right? Or one time I was driving and there was a snowstorm, I was snowed in on the road for two days. That is another moment that you have to face the facts [about living with HIV]. Because if that would happen again and I didn’t bring enough medication… I’m screwed because of that dependency [on ART]. Every single day I am faced with that dependency. [male, 61, gay, living with HIF for 20 years]
	Being constantly dependent on ART	12	I switched therapy already like eight-nine times. And with any of those, I never had a perfect combination of medication without experiencing side effects and additional problems. It is still far from perfect, and I see that it's like a huge improvement compared to twenty years ago. The side effects are less and less and there is less damage for the body… [female, 42, heterosexual, living with HIV for 7 years]
	Being constantly dependent on ART	13	Yeah, I’m always worried. Definitely now, with COVID-19… I’m afraid the medication will become scarce. That we are in a situation that HIV medication is no longer available. The same with a war situation… And with COVID-19, that they thought that HIV medication would help with the treatment [of COVID] … Yeah, I’m sure it will lead to the scarcity of [HIV] medication. [female, 53, heterosexual, living with HIV for 10 years]
	Being constantly dependent on ART	14	With the COVID-19 vaccine… I’m thinking “that will probably influence my medication” because it hasn’t been tested that much and it doesn’t exist that long. They’ve developed it so fast, have they tested it on different people and medication users? [female, 43, heterosexual, living with HIV for 16 years]
	Continuous struggles of disclosing HIV	15	They [family] don’t know, it [the relationship] hasn’t changed [male, 30, gay, living with HV for 4 years]
	Continuous struggles of disclosing HIV	16	I told four people in the first year [about HIV]. But I did notice over the course of that year, an increase in tension within me. Because I don’t like it if I can’t talk about something, so that resulted in me not talking to my family anymore. And if friends came over, I had a complete panic attack because I felt that they could tell by looking at me [that I had HIV] Everything just got worse after that… [male, 27, gay, living with HIV for 4 years]
	Continuous struggles of disclosing HIV	17	Like, if you have a date, for example, I’m thinking “I should tell them, I don’t want to keep a secret” or something. That is something that I struggle with now I have HIV myself. “Shall I tell them immediately?” Then you know almost certainly that sex won’t happen unless this person is very well informed. But 9 out of 10 times people asked about it on dating apps: “What is your HIV status?” or they asked: “Are you clean?” And then, should I say: “No, I have HIV”? You just know that they will block you. So, what should I do? Should I be honest? Or not share it [having HIV]? I can’t bring the other person in danger. It sounds like a lot… but in every situation I would think about it again. [male, 31, gay, living with HIV for 6 years]
	Ongoing fear related to HIV	18	Yes, I am sad about it [HIV]. But that's more because of the situation. Not because it [HIV] happened but because of the situation that I once lost love because of it. And that makes it [HIV] difficult… It made me live a little less intensely or live a little more peacefully. But has HIV changed me? Well, it only plays a role in my social contacts with women or relationships. It really made a significant change there… Starting a relationship, because then you have to tell that you have it [HIV]… I am careful who I choose to talk about it [HIV]… [male, 45, heterosexual, living with HIV for 4 years]
	Ongoing fear related to HIV	19	I grew up believing that it was not possible to have sex with someone without a condom because there was always something [HIV] hanging over your head… Now, of course, a huge step has already been taken with PrEP. But it remains true that sex entails a certain kind of danger. That can be very fascinating, on the one hand, but, of course, it is also an enormous limitation in your experience of sexuality, on the other hand. Plus, you've grown up with fear of that disease [HIV]. That means even though you’ve done it safe, there’s always a moment when you’re testing [for STI’s and HIV] …there is always a certain kind of stress factor in it. [male, 41, gay, key population]
	Ongoing fear related to HIV	20	We would like to have children and my partner finds… [sex] without a condom uncomfortable [because of HIV]. So that means we have to try with a jar, a syringe, and ovulation tests. And no… that’s not as fun. [female, 39, heterosexual, living with HIV for 11 years]
	Unyielding HIV-related self-stigma and stigma	21	And because of ignorance, I feel that people are also scared. Yeah, when they know that I have HIV… That is also when I feel stigma. For example, I have a friend with a small kid. And the kid asked me to blow her balloon… And I took the balloon in my hand. This friend of mine was the father of the kid. He took it immediately away from my hands. And said, “no don’t worry, I will do it”. I felt he was scared that I might blow with my mouth and then maybe the kid would also blow it and touch the balloon where I touched it. I know there are two types of stigmata. Like one is external and one is self-stigma, but [in examples such as these] they both are feeding each other because when I see this kind of reaction from people around me then I feel both. [female, 42, heterosexual, living with HIV for 7 years]
	Unyielding HIV-related self-stigma and stigma	22	That was at the beginning, when I would see my veins on my hands and I would think: “geez, there’s really deadly poison running through here”. [female, 57, heterosexual, living with HIV for more than 20 years]
	Unyielding HIV-related self-stigma and stigma	23	The strange thing is, I notice these [stigmatizing attitudes] especially in healthcare. I would come to the hospital for something else and then I would have to have blood drawn and the nurse would be messing around. So, I would say, “Is it not working?” “No”, she says, “I never do it with gloves on”. “Why are you doing it with gloves on now?” “You have HIV” … I take medication, I can no longer transmit the virus. And you think, they should know that if they work as a nurse in a hospital, right? And, I mean, I can speak up, and I can explain it at that moment, but it’s not fun. And I can imagine that there are people who do experience stigma, for whom it would be a very traumatic moment, when someone else treats you differently because you have HIV. So, you are always providing information while you would think, people with such a profession should know better, right? Someone [not in the medical world] who has never had to deal with it [HIV], is not aware of the latest developments. I get that. But someone in the medical world you would think differently [female, 58, heterosexual, living with HIV for more than 20 years]

#### Being constantly dependent on ART

The dependency on ART was explained by both participant groups as a negative influence because HIV made a person perpetually dependent on ART to live a long and healthy life. The reduction of spontaneity due to the dependency on ART was another aspect that participants perceived as a negative influence on their QoL: Table 3*Quote 11*. In addition, interviewees from both groups mentioned that the side effects of ART negatively influenced their QoL. PHIV and key populations acknowledged an ambivalence by describing that ART improved drastically over the last decades, but this improvement was not always experienced in their current QoL. Some PHIV explained this in the form of side effects of ART: Table 3*Quote 12*. The dependency on ART caused both PHIV and participants from key populations to worry during the COVID-19 pandemic because they were concerned about the supply of ART that could undermine their ability to live normal lives (Table 3*Quote 13*). Participants with HIV also worried that the potential COVID-19 vaccines would reduce the effectiveness of ART (Table 3*Quote 14*).

#### Continuous struggle of disclosing HIV

Some participants PHIV and key populations described to experience a continuous struggle related to discussing HIV in new relationships. PHIV described situations when they decided to disclose or not to disclose living with HIV to family and friends. In instances when they opted for the latter, they strived for normality in their existing relationships: Table 3*Quote 15*. PHIV who did not disclose their HIV status to family and friends struggled with their decision. They indicated to feel alienated from family and friends and being distressed when they had not disclosed yet that they had HIV (Table 3*Quote 16*). In addition, some PHIV and participants from key populations expressed to feel obligated to discuss HIV in dating life to prevent feelings of dishonesty: Table 3*Quote 17*.

#### Ongoing fear related to HIV

Some interviewees from both groups mentioned ongoing fear related to HIV. The predominant influence of HIV was described as a reason for fear in several domains: fear of rejection among PHIV and fear of contracting HIV among members of key populations.

First, some participants with HIV mentioned that their fear of rejection made it difficult to disclose HIV to others. While several participants with HIV had experienced rejection due to HIV, they did not perceive this as a loss for themselves. Other PHIV who experienced rejection due to HIV were more afraid of rejection in the future. This fear led them to be more careful about discussing HIV (Table 3*Quote 18*). Second, several MSM without HIV expressed ongoing fear of contracting HIV. They shared to be scared of contracting HIV and mentioned it as the main reason for their current preventive behavior described as “*I always use condoms*” [male, 38, bisexual, living with HIV for 7 years] and “*I don’t have anal sex*” [male, 44 gay, key population]. Therefore, they described sex as both exciting and frightening at the same time (Table 3*Quote 19*). Several women and men with HIV talked about fear of transmitting or acquiring HIV when pregnancy was considered between partners: Table 3*Quote 20*.

#### Unyielding HIV-related self-stigma and stigma

Several participants from both groups described various experiences of HIV-related self-stigma and stigma. One of the aspects in which self-stigma was revealed was related to the communicable nature of HIV that made participants “*feel dirty” [male, 33, gay, living with HIV for 5 years]*. The moment other people reacted anxiously about HIV was explained as reinforcing self-stigma (Table 3*Quote 21*). The communicable nature of HIV was also often mentioned by long-term survivors who described a “*back then*” situation: Table 3*Quote 22*. In addition, many interviewees from both populations described to experience stigmatization by the general population. Especially interactions with health professionals, such as surgeons, nurses extracting blood for analyses, dentists, and pharmacists, were experienced as stigmatizing by PHIV (Table 3*Quote 23*).

## Discussion

Our thematic analysis demonstrated the current influence of HIV on the QoL of PHIV and key populations in the Netherlands. Although the clinical management of HIV was considered to have changed in the last decades, and ART, PrEP, and U = U did lead to positive meaningful experiences related to HIV, both groups regarded the multifaceted and predominant influence of HIV on their QoL as too broad to be improved by clinical management alone.

Our results describe positive meaningful experiences of HIV among both participants groups. A review of the qualitative literature by Herron et al.^16^ illustrated similar findings among women with HIV. Many men and women with HIV in our study noted being grateful for being physically fit, and they attributed supporting and receiving support from a community to be a positive influence of HIV on their QoL. A possible explanation for positive adjustment to living with HIV resulting in a positive influence on the QoL may be a personal process of acceptance of HIV^33,34^. For example, participants who accepted living with HIV seemed to be motivated to support other people with HIV. Providing guidance to PHIV and key populations vulnerable to HIV in accepting and positively adjusting to their HIV status may reduce the need to conceal HIV and make it easier to disclose HIV, and ultimately result in a positive influence on their QoL.

Being diagnosed with HIV or being vulnerable to HIV in the pre-ART era may have caused a significant burden in the past^5,16,35–39^. The predominant and negative influence of HIV shared by participants in our study was described to have improved due to ART, PrEP, and U = U, but it did not disappear^5,33,40–42^. Improvements in clinical management in the ART era meant new discomforts for participants, such as the side effects of ART. Improved clinical management in the ART era may have significantly improved the clinical outlook and many aspects of the QoL of long-term survivors. This improvement of the negative influence of HIV may have not been experienced by people diagnosed more recently in the ART era. Unlike long-term survivors, recently diagnosed individuals or young individuals from key populations vulnerable to HIV have not experienced fear of HIV, the amount of medication, and its side-effects to the same extent as did long-term survivors diagnosed in the pre-ART era. Therefore, PHIV diagnosed in the ART era may not be used to the burden of the early HIV treatment and its severe side effects. Thus, participants from key populations and diagnosed with HIV in the ART era may have shared many negative influences related to ART that long-term survivors from the pre-ART era would not.

MSM participants with and without HIV revealed that the influence of HIV has changed due to PrEP in recent years because it provides more control over their sexual health and freedom. This changed influence was linked to the reduction of fear of HIV acquisition and to the increasing autonomy over their preferred choice of HIV prevention (e.g., use condoms or PrEP)^22,43,44^. Some studies found that PrEP may reduce stigmatization towards PHIV among MSM^22^, especially when selecting potential sex partners. We found that PHIV and key populations still experienced stigmatization and discomfort, despite PrEP, e.g., when discussing HIV with partners. Currently an increased openness about one’s HIV status was perceived difficult due to the limited knowledge about HIV and biomedical interventions in HIV management in the general population. The lack of media attention about these matters may explain the continued experiences of stigmatization^45,46^. Efforts in society, such as increased positive media coverage may improve the experience of discussing HIV. Additionally, we hypothesize that PrEP may cause a positive shift in moving HIV from a private to a more public matter^35–39,47–51^. If PrEP use is normalized in clinical practice and beyond, and knowledge about PrEP is universal, PHIV may feel that concealing HIV is unnecessary which could further reduce stigmatization they experience when discussing HIV with partners^35–39,47–51^.

In addition, the negative influence of HIV described by participants was mainly related to social factors and interpersonal relationships, as also noted by Andersson et al. who showed that these factors have changed in the last decades. In early years of the HIV pandemic when medication was not available, HIV was linked to activism and was a matter of constant public discussion^5,16,35^. Activism was necessary to make sure that HIV became a priority and to accelerate the development of effective HIV treatment. After the ART became available, HIV might have started to shift to being a private matter, leaving PHIV to deal with HIV alone. What started as a social and open discussion, might have changed to an individual and secret problem. During the ART era, PHIV started concealing HIV to normalize their life and separate it from other areas of their life. This normalization of their life may explain why participants described to find it difficult to disclose that they have HIV^41,52,53^. This concealment may have resulted in loneliness and stress, unlike the situation with other chronic diseases^35–39,47–50^.

Finally, our results showed that improvements in treatment and prevention of HIV were able to change the influence of HIV on the QoL experienced by PHIV and key populations. We hypothesize that new treatment and prevention options such as, long-acting ART or an HIV cure may further influence how HIV is experienced by PHIV and key populations^54–59^. Kerrigan et al.^54,55^ and Simoni et al.^59^ described that long-acting ART has the potential to remove the daily reminder of ART, and, in light of our findings, we hypothesize that this type of ART may help further improve the QoL of PHIV and key populations^6^. Ultimately, while new treatment and prevention options may change the experienced influence of HIV on the QoL^54,55,59^, only an HIV cure that would eliminate HIV from the body is believed to be the way to remove the multifaceted and predominant influence of HIV completely^6^.

### Strengths and limitations

This study has several strengths. While previous studies focused mainly on exploring the burden of HIV among PHIV, our study explored the influence of HIV among both PHIV and key populations, particularly highlighting the presence of positive meaningful experiences related to HIV that received little attention in prior studies. By triangulating data analysis with three researchers [K.A.G.J.R., L.d.G. and S.C.J.M.V.], we were able to strengthen the credibility, transferability, dependability, and confirmability of our findings^60^. There are also some limitations. Due to the qualitative nature of our results, our study generated themes, issues, perspectives, and ideas that have societal relevance, but it was not meant to provide an external representative quantification^61^. Demographics, biomedical interventions, and time may play a role in QoL and stigma experienced now, compared to past experiences. Despite some observed variation among participants in age, education, time since diagnosis, and gender, we cannot assess differences in reported findings and demographics among participants due to the qualitative nature of our study. Additional research may explore how HIV transmission routes affect the QoL of PHIV and key populations. Although we aimed for maximum variation in our participants characteristics, our entire sample included mostly highly educated persons and MSM among key populations. Other groups of key populations vulnerable to HIV, such as sex workers, may offer different perspectives. We suggest further research among these groups and research in other geographical settings such as Sub-Saharan Africa to generate themes, issues, and perspectives relevant for other contexts. Further research is needed to investigate the generalizability of the themes explored and to address possible differences in the perceptions about the influence of HIV on QoL, for example among different groups of key populations.

## Conclusion

In conclusion, despite all the advances in biomedical interventions in treatment, care, and prevention the multifaceted and predominant influence of HIV remains, especially on the societal and interpersonal levels. This influence requires further attention and support to improve the QoL of PHIV and key populations vulnerable to HIV. It remains important to support HIV community organizations in their efforts to reduce stigmatization and to continue improving biomedical interventions for HIV as these positively influence the QoL or further reduce the negative influence of HIV on the QoL of PHIV and key populations.

## Supplementary Information

Supplementary Tables.

## Data Availability

Only the authors have access to the raw data quoted in this study due to confidentiality. Any reasonable request for access to material relating to the study can be made directly to the corresponding author, who will negotiate information sharing on a case-by-case basis.

## Abbreviations

ACS: Amsterdam cohort studies
ART: Antiretroviral treatment
MSM: Men who have sex with men
PHIV: People with HIV
PrEP: Pre-exposure prophylaxis
UMC Utrecht: University Medical Center Utrecht
QoL: Quality of life
COREQ: Consolidated criteria for reporting qualitative studies
U = U: Undetectable equals untransmittable
