# Supplementary material for: The experienced positive and negative influence of HIV on quality of life of people with HIV and vulnerable to HIV in the Netherlands
Source: Sci Rep. 2022 Dec 19;12:21887. doi: 10.1038/s41598-022-25113-5 (PMC9761623; doi:10.1038/s41598-022-25113-5)
Supplement: Supplementary file 1 — Supplementary Tables. [file 41598_2022_25113_MOESM1_ESM.pdf]

THE EXPERIENCED POSITIVE AND NEGATIVE INFLUENCE OF HIV ON QUALITY OF LIFE OF PEOPLE WITH HIV AND VULNERABLE TO HIV IN THE NETHERLANDS

Kim A.G.J. ROMIJNDERS[1, *],

Laura DE GROOT[1],

Sigrid C.J.M. VERVOORT[1],

Maartje BASTEN[1],

Berend J. VAN WELZEN[2],

Mirjam E. KRETZSCHMAR[1],

Peter REISS[3,4],

Udi DAVIDOVICH[4,5],

Maarten F. SCHIM VAN DER LOEFF [3,6],

Ganna ROZHNOVA[1,7]

SUPPLEMENTARY TABLE I

Supplementary Table 1 Consolidated Criteria for Reporting Qualitative Studies [30]

Domain 1: Research team and reflexivity		Pages
Personal Characteristics		
1. Interviewer/facilitator	Which author/s conducted the interview or focus group? K.A.G.J.R. *conducted all the interviews.*	9
2. Credentials	*PhD*	n/a
3. Occupation	*Assistant Professor*	n/a
4. Gender	*Female*	n/a
5. Experience and training	*Extensive training and over 5 years of experience in qualitative research.*	9
Relationship with participants		
6. Relationship established	Was a relationship established prior to study commencement? *No*	8
7. Participant knowledge of the interviewer	What did the participants know about the researcher? *The researcher introduced herself at the start of the interviews as a researcher with extensive experience in qualitative research.*	8-9
8. Interviewer characteristics	What characteristics were reported about the interviewer/facilitator? e.g., Bias, assumptions, reasons, and interest in the research topic<br><br>*“An academic health behavioral scientist.”*	9

Domain 2: study design		
Theoretical framework		
9. Methodological orientation and Theory	What methodological orientation was stated to underpin the study? e.g., grounded theory, discourse analysis, ethnography, phenomenology, content analysis<br><br>*A thematical analysis was performed [32, 72].*	9
Participant selection		
10. Sampling	How were participants selected? e.g., purposive, convenience, consecutive, snowball<br><br>*Participants were purposely sampled with the help of the partners at the Amsterdam Cohort Studies, the AGE$_h$IV Cohort Study, the infectious diseases outpatient clinic of the UMCU, and the Dutch HIV Association*	8
11. Method of approach	How were participants approached? e.g., face-to-face, telephone, mail, email<br>*Participants were contacted by telephone by K.A.G.J.R. to schedule an interview.*	8-9
12. Sample size	How many participants were in the study?<br>*42*	10

13. non-participation	How many people refused to participate or dropped out? Reasons?<br>*Of those who showed interested in the study, 2 dropped out. No reason was provided.*	10
Setting		
14. Setting of data collection	Where was the data collected? e.g., home, clinic, workplace<br>*Due to Covid-19 restrictions, 31 interviews were conducted online and 11 were conducted face-to-face.*	8-9
15. Presence of non-participants	Was anyone else present besides the participants and researchers?<br>*No.*	n/a
16. Description of sample	What are the important characteristics of the sample? e.g., demographic data, date<br>*Of the 44 scheduled interviews, 42 were conducted. Twenty-nine of the participants were living with HIV, and 13 belonged to key populations. Nine of the 42 participants were women, of whom one identified as a transwoman, and ages ranged from 24 to 72 years. Overall, many PLHIV and participants without HIV shared experiences about the multifaceted influence of HIV on their QoL that were described as predominant and negative or positive and meaningful.*	10
Data collection		

17. Interview guide	Were questions, prompts, guides provided by the authors? Was it pilot tested?<br>*Semi-structured in-depth interviews were conducted using an interview topic guide (table 1).*	8, 40
18. Repeat interviews	Were repeat interviews carried out? If yes, how many?<br>*No.*	n/a
19. Audio/visual recording	Did the research use audio or visual recording to collect the data?<br>*All interviews were audio recorded.*	8
20. Field notes	Were field notes made during and/or after the interview or focus group?<br>*Notes were taken during the interviews to describe nonverbal communication.*	9
21. Duration	What was the duration of the interviews or focus group?<br>*45 to 90 minutes.*	8
22. Data saturation	Was data saturation discussed?<br>*The sampling was scheduled to stop after inductive thematic saturation had been reached [30].*	8
23. Transcripts returned	Were transcripts returned to participants for comment and/or correction?<br>*No.*	n/a
Domain 3: analysis and findings		
Data analysis		

24. Number of data coders	How many data coders coded the data?<br>*Three researchers*	8-9
25. Description of the coding tree	Did authors provide a description of the coding tree?<br>*No.*	n/a
26. Derivation of themes	Were themes identified in advance or derived from the data?<br>*No themes were identified in advance.*	9
27. Software	What software, if applicable, was used to manage the data?<br>*NVivo version 12.*	8-9
28. Participant checking	Did participants provide feedback on the findings?<br>*No.*	n/a
Reporting		
29. Quotations presented	Were participant quotations presented to illustrate the themes / findings? Was each quotation identified? e.g., participant number<br>*Yes.*	10-22
30. Data and findings consistent	Was there consistency between the data presented and the findings?<br>*Yes.*	10-22
31. Clarity of major themes	Were major themes clearly presented in the findings?<br>*Yes.*	10-22

32. Clarity of minor themes	Is there a description of diverse cases or discussion of minor themes?<br>*Themes and sub-themes are discussed in the results.*	10-22

SUPPLEMENTARY TABLE II

Supplementary Table II Stages of thematic analysis

Stage	Description
1) Familiarization of data	K.A.G.J.R. and L.d.G. transcribed the data, reviewed the transcripts, and noted down initials ideas after each cycle was completed.
2) Generating initial codes	K.A.G.J.R. and L.d.G. coded the data using NVivo after each cycle was complete. This way, data coded were automatically grouped per code.
3) Searching for themes	After all cycles of in-depth interview were completed, K.A.G.J.R. and L.d.G. applied specific codes to themes and sub-themes to cluster all relevant data among themes. After this, more patterns and repetition were investigated by generating thematic maps.
4) Reviewing themes	K.A.G.J.R. and L.d.G. reviewed all codes gathered for specific themes to identify patterns. They re-read a selection of random transcripts to examine validity of the themes and sub-themes generated. They checked the

	validity of the themes to explore whether they represented the data.
5) Defining and naming themes	K.A.G.J.R. and L.d.G. refined the themes and sub-themes continuously to make sure the overall story of the data was expressed. This led to clear ideas and concepts for each theme and sub-theme, which were peer reviewed by S.C.J.M.V (a qualitative research expert).
6) Producing the report	Quotes were selected, directly derived from our data, to provide examples of the themes and sub-themes in the report of this study.
